# John Rex: the case for investment in antimicrobials

**DOI:** 10.2471/BLT.23.030623

**Published:** 2023-06-01

## Abstract

John Rex talks to Gary Humphreys about the challenges faced in developing and bringing to market new antibiotics.

*Q: Despite increasing concern about antibiotic resistance, very few new antibiotics are emerging from the R&D pipeline. What is holding back their development?*

A: Well first, it’s probably worth stating why that matters. Over time, and as a result of adaptation driven by natural selection, certain microorganisms have become resistant to the antibiotics that previously either killed them or prevented them from reproducing. This includes *Klebsiella pneumoniae* and *Escherichia* *coli*, some strains of which have become resistant to all or nearly all available antibiotics. It’s estimated that bacterial drug resistance kills around 1.3 million people every year directly, and contributes to the deaths of another 5 million. To tackle that problem, we need antibiotics that work differently, and we are not currently developing them. The various reviews done of compounds in the R&D pipeline suggest that many are modifications of existing drug classes, and will not be sufficient to address antimicrobial resistance.

As to why the pipeline is so thin, it comes down to the fact that the antibiotic business is not profitable under normal market conditions. It takes 10–15 years and sometimes longer to develop a new antibiotic at a total cost that exceeds 1 billion US dollars (US$) when you include inevitable failures. If you are lucky enough to arrive at a safe and effective product, you then find that there is a limit to the sales you can achieve because clinicians are going to use it as sparingly as possible. And rightly so: the more an antibiotic is used, the more quickly microorganisms develop resistance to it. This is different to every other class of drug, which may get replaced by better drugs, but don’t themselves lose their therapeutic power. Add to this all your other costs, such as the need to manufacture fresh stock on a regular basis, and you’re struggling. The best current estimate is that it costs around US$ 350 million to complete post-approval work and manufacture the compound during its first 10 years on the market, which means you need to generate US$ 35 million a year just to break even on a cash-flow basis. So, bottom line, because companies struggle to recover their costs, let alone make a profit, they have tended to drop out of the market.

“Innovation is not the problem; the market model is the problem.”

In the 1980s there were something like 18 multinational companies committed to antibiotic research. Today, there are only a handful. The space left by those companies has been filled by smaller biotech companies, which today account for around 80% of the investigational antibiotics that have the potential to become tomorrow’s big weapons in the fight against antibiotic resistance. Unfortunately, most of those companies are struggling financially, and there are plenty of examples of small companies that have come up with promising new drugs and pushed through all the clinical trial and regulatory challenges, only to go bust after they have launched their product.

*Q: Can you give some examples?*

A: A recent example is Nabriva Therapeutics, which spun off from Sandoz’s antibiotic research and development (R&D) operations and discovered a drug called lefamulin in 2006. The drug can be taken by mouth or injection, and is used to treat adults with community-acquired bacterial pneumonia. Around a million people are hospitalized with that disease in the USA each year, some 50 000 of whom die, and there are of course many more cases worldwide. So, it had the potential to be a useful, impactful drug, and in clinical trials was shown to be comparable in clinical outcomes to moxifloxacin, a widely used antibiotic. Moreover, lefamulin was shown to have in vitro activity against other pathogens including *Streptococcus pneumoniae* and methicillin-resistant *Staphylococcus aureus*. So, all in all, an interesting drug. It was approved for medical use in the USA in August 2019 and in the European Union in July 2020. Nabriva launched the drug as Xenleta, but couldn’t generate enough sales, and announced that it was winding down its business in January of this year. Other examples include the antibiotic firms Melinta Therapeutics, Aradigm and Achaogen, which all declared bankruptcy in 2019. So, it can be argued that innovation is not the problem; the market model is the problem.

*Q: How can we address this?*

A: We need to look at the way we assign value to antibiotics, getting past the notion that value has to be a function of use. I find that a helpful way to get this idea across is to compare antibiotics to fire extinguishers. The analogy isn’t perfect since fires do not become resistant to extinguishers, but there are some interesting points of comparison. Like fires, infectious disease outbreaks occur abruptly and spread rapidly. Like fire extinguishers, antibiotics appear to have zero value until an outbreak occurs, at which point, like fire extinguishers, they become enormously valuable. I’d also add that, like fire extinguishers, antibiotics don’t just appear in the home: they have to be developed, manufactured, maintained and properly used.

It is often pointed out that the high casualty rate of the 1918 influenza pandemic wasn’t due to the virus itself, but rather to the lack of antibiotics to treat pulmonary infections. A similar case can be made for the coronavirus 2019 (COVID-19) pandemic: for lack of a good fire extinguisher, the world caught fire. The beta coronaviruses that cause upper respiratory illness have been known for 20 years. If someone had developed a drug that was effective in treating such illnesses, we could have nipped the outbreaks in the bud, saved millions of lives, and avoided the shutdown of the global economy. Nobody developed that drug because there was no market for it. And if a company had developed it, the company would have made some money when the pandemic arrived, but the real value of what they had saved the global economy would not have been recognized. We need to get our heads around the idea that something we don’t use often nevertheless has significant value, and indeed question the notion of use itself. If I asked you if you used a fire extinguisher today, you’d probably say, “No.” But I’d have to disagree: just by having one on the wall, you are using it.

*Q: How do you get people to pay to develop something they may never use?*

A: In the same way people pay for insurance: in instalments. This approach has been debated for some time, and the consensus view is that we need some form of subscription, whereby the beneficiaries of the product – let’s say a government on behalf of its population – makes regular payments to companies that successfully develop a needed new antibiotic. By doing this you effectively delink the companies’ revenue stream from sales of their products. They no longer need a regular stream of sales, relying instead on the subscription, which acts as a ‘pull incentive’. This approach is also great for antibiotic stewardship, since the company no longer needs use-based sales to stay in business. Of course, you have to come up with criteria for picking the antibiotics purchased in this way. Some countries are now considering the introduction of such subscription schemes, led by the United Kingdom, where the National Health Service has agreed to pay Pfizer and Shionogi a fixed fee of £10 million (US$ 12.5 million) a year for ten years for two antibiotics. As a pilot, the United Kingdom’s initiative offers a first opportunity to see a delinked pull incentive at work and can serve to inspire other initiatives.

“For lack of a good fire extinguisher, the world caught fire.”

*Q: But is US$ 12 million enough to incentivize R&D, given the costs you cited?*

A: You have to look at that amount in the broader context. Numerous studies, including a recent one by the United States Government Accountability Office, suggest that providing a global total of US$ 1– US$ 4 billion over a 10-year period after approval by regulatory authorities is sufficient. The best current estimate to date is from work by Kevin Outterson, and suggests around US$ 3.1 billion for an effective, fully delinked subscription. If other major economies contributed amounts similar to the United Kingdom on a proportionate basis, for example as a percentage of gross domestic product, the economics of new antibiotic development would become similar to those in the development of new cancer therapies.

Encouragingly, all of the G7 countries are seriously considering pull incentives. In the USA, the PASTEUR (Pioneering Antimicrobial Subscriptions to End Up-surging Resistance) Act was recently introduced into the 118^th^ United States Congress. The act is a bipartisan piece of legislation aimed at incentivizing innovative drug development, targeting the most threatening infections and supporting appropriate use of antibiotics. Drafted over two years ago, it has now been thoroughly debated and there is growing pressure to get it passed this year. If it does pass, it will be a game-changer in the antibiotic innovation space. Meanwhile, the Canadian government is putting together a report on pull incentives; Japan has announced its first steps towards introducing pull incentives; and the European Commission has very recently proposed that pull incentives be implemented using transferable data exclusivity vouchers as a bridge to by-country delinked procurement models. It is very exciting to see such a wide range of approaches to the problem *–* there’s obviously a strong recognition that something has to be done about AMR!

*Q: Are you optimistic that all this discussion will feed through into implementation?*

A: I am. You have to remember that 10 years ago we did not have much activity at this level. There were significant initiatives like the Infectious Diseases Society of America’s “Bad Bugs, No Drugs!” and “10×20” initiatives, but momentum has really been gathering since 2016, not just with new initiatives such as the Global Antibiotic Research & Development Partnership which was set up by WHO and the Drugs for Neglected Disease initiative that year, but with increased prioritization by global political leaders. The 2016 United Nations General Assembly (UNGA) on antimicrobial resistance is the clearest expression of this priority. The topic will be on the UNGA agenda again in 2024, with a focus on committing to new targets and practical steps to address the problem. What could be more practical than developing and installing the fire extinguishers we need to tackle the next outbreak? And the time to start making them is now!

